# Determinants of timely access to Specialized Mental Health Services and maintenance of a link with primary care: a cross-sectional study

**DOI:** 10.1186/s13033-021-00507-6

**Published:** 2021-12-04

**Authors:** Carlos Alberto dos Santos Treichel, Ioannis Bakolis, Rosana Teresa Onocko-Campos

**Affiliations:** 1 Department of Collective Health, School of Medical Sciences, University of Campinas, St. Tessália Vieira de Camargo, 126. Cidade Universitária Zeferino Vaz., Campinas, SP Zip code 13083-887 Brazil; 2 Department of Biostatistics and Health Informatics, Health Services and Population Research Department, Institute of Psychiatry, Psychology and Neuroscience, King’s College London, London, UK

**Keywords:** Pathways to Care, Mental Health, Community Mental Health Services, Primary care

## Abstract

**Background:**

Although access to specialized services is one of the main components of the study of paths to mental health care worldwide, the factors related to the continuity of the patient’s link with Primary Care after admission to a Specialized Mental Health Services still need to be explored in greater depth. Thus, this study aimed to evaluate the determinants of timely access to Specialized Mental Health Services (outcome 1) and maintenance of a link with Primary Care after patients’ admission (outcome 2).

**Methods:**

This is a cross-sectional study, conducted with 341 users of Specialized Mental Health Services at outpatient and community level in a medium-sized city in Brazil between August and November 2019. Associations between the outcomes and the other variables were explored with the use of Poisson regression models with robust variance estimators.

**Results:**

Factors positively associated with timely access were the diagnosis of psychosis or psychoactive substance misuse. The inversely associated factors with this outcome were higher income, having their need for mental health care identified in an appointment for general complaints, having been referred to the current service by Primary Care, having attended the current service for up to 3 years and delay until the first appointment (in a previous service). Regarding the maintenance of a link with Primary Care, factors positively associated were being referred to the current service by Primary Care or private service and receiving visits from Community Health Agents. The inversely associated factors with this outcome were male sex, being employed, having a diagnosis of psychosis or psychoactive substance misuse, and a greater perception of social support.

**Conclusions:**

In addition to individual factors, factors related to the organization of services and the referral between them stood out in influencing both the access and maintenance of the patients’ link with Primary Care. Thus, this study reinforces the idea that integration between Primary Care and Specialized Mental Health Services should be strengthened, both to reduce waiting times for between-service referrals and benefit of care continuity.

## Background

The study of pathways to mental health care is an important means of understanding how health systems work and which parts of the pathways that need to be targeted by initiatives to support timely access to specialized programs. A systematic review of pathways to mental health care in 23 countries [1] pointed out that considerable variations of pathways to mental healthcare across different countries still exist and the role of Primary Care doctors and social networks still represent an unsolved issue.

Data obtained by pathways to mental health care studies may play a crucial role in formulating mental health policies and in the organization of psychiatric services [2]. However, this type of study is still emerging in low and middle-income countries, especially in Latin America. While studies are found in Cuba and Mexico [3], a systematic review conducted in Brazil [4] indicated that few quantitative studies have addressed specific stages of pathways to mental health care in the country.

It is estimated that 6.9 million Brazilians (3.3%) present severe and persistent mental health disorders requiring intensive and continuous mental health care [5], while an additional 30.2 million (14.5%) present mild or moderate mental health disorders requiring occasional treatment in specialized services [6]. Despite that, as observed in other countries, there is still an important treatment gap, with the lack of trained professionals to provide mental health care in Primary Care and the low integration with the mental health network being the main challenges [7].

A study conducted in the largest of Brazil’s metropolitan areas found that in the last 12 months, just one in five adults with any psychiatric disorder used a mental health service, and only one in ten consulted a psychiatrist [8]. This study also showed that specialized services delivered more appointments than Primary Care and that a greater proportion of individuals received a minimum threshold of adequacy in specialized care as compared to Primary Care.

Although access to specialized services is one of the main components of the study of pathways to mental health care, another aspect that needs to be explored in greater depth concerns to the continuity of the patient’s link with Primary Care. Studies have shown that after accessing specialized services, many patients lose their link with Primary Care [9, 10], which can lead to neglect of physical health conditions and poor clinical outcomes [11].

It is known that several factors may influence the access to specialized care, such as: age, gender, ethnicity, beliefs about mental illness, family involvement in help seeking, geographical characteristics and aspects related to the arrangement of the systems of care (e.g. referral route and service structure) [12]. On the other hand, the factors related to the maintenance of a link with Primary Care by users who accessed specialized mental health services remain unknown.

Recognition of these factors is especially relevant in the Brazilian context, since although the country’s health system operates in the stepped care model, its specialized outpatient mental health services provide access through several ways, including spontaneous demand [4].

In the face of that, the aim of the current study is to fill this gap in the literature and evaluate the determinants both of timely access to Specialized Mental Health Services and the maintenance of a link with Primary Care in a medium-sized city in the state of São Paulo, Brazil.

## Methods

### Study design and sample

We conducted a cross-sectional study from August to November 2019 with users from three Outpatient Mental Health Care Services in the municipality of Itatiba, located approximately 80 km from the state capital of São Paulo. This city is part of the Metropolitan Region of Campinas, and according to the last census, the estimated population in 2019 was 120,858 inhabitants [13]. The city has 19 Primary Care services, and in addition to hospital and emergency services, there are three Outpatient Services providing mental health care to the population. These services are the Psychosocial Care Center II (PCC-II), the Psychosocial Care Center for Psychoactive Substance Misuse (PCC-PSM), and an Outpatient Clinic. According to data collected in a prior survey, 1958 users were attending these services during the study period.

The selection of participants was carried out by simple random sampling. From a list of outpatient service users, the medical records of 386 users who met inclusion criteria were selected, contacted by telephone, and invited to take part in the study. Inclusion criteria were being >18 years of age and being enrolled in the service for at least one month. Individuals were excluded from the study if they reported having received a diagnosis of intellectual disability, which could compromise their ability to answer the questionnaire.

Contact with participants was initially made by telephone, at which time they were informed of their right to not participate in the research if they did not wish to do so, to cease participating in the research at any time, and to remain anonymous. Data collection by questionnaire was carried out in the service facilities between August and November 2019 and was scheduled to take place on days when patients were already attending the service for appointments or to participate in some other activity.

The construction of the questionnaire was based on a literature review that considered previously established potential determinants regarding access to and use of health services. The questionnaire, consisting of 64 questions, also included questions about sociodemographic characteristics, therapeutic offers received, health conditions and satisfaction with the services. The validation of the questionnaire was carried out in a series of workshops that brought together researchers, workers, and patients from specialized mental health services.

Questionnaires were administered by six psychology undergraduate students, one physician, and one social worker. These individuals were included in the study through a selective process and received training in the application of the questionnaires.

Before applying the questionnaire, the study consent form was read aloud to the participant by the questionnaire’s administrator. Participants signed the consent form after being afforded the opportunity to have any questions answered.

### Measures

Timely care in Specialized Mental Health Service was defined as the user having been attended within 15 days following referral. To measure this outcome, the following question was used: “*How long did it take from your referral (or self-referral) to this service until your first appointment here?*”.

Maintenance of a link with Primary Care after treatment at a Specialized Mental Health Service was defined as at least one contact with Primary Care services in the last 6 months, following the initial visit to the Specialized Mental Health Service. To measure this outcome, the following question was used: “*How long has it been since your last appointment in Primary Care? (For any health problem or need, even if it is not related to your mental health)*”.

### Covariates

The independent variables included in this study were: sex (female; male); ethnicity (white; mixed race; black); age (18–30; 31–45; 46–60; 61+); schooling (0 to 4 years; 5 to 8 years; 9+ years); paid work status (unemployed; employed); per capita income (< 0.5 minimum wage; 0.5 to 1 minimum wage; > 1 minimum wage); diagnosis (affective and neurotic disorders; psychosis; psychoactive substance misuse; as yet undiagnosed); identification of the need for mental health care (in a crises situation; self-diagnosis followed by spontaneous demand; indicated by family and friends; in an appointment for general complaints); delay until first appointment (up to 7 days; up to 30 days; up to 90 days; up to 1 year; > 1 year); first appointment service (Primary Care; emergency or hospital services; outpatient clinic; Psychosocial Care Centers; private services); referral source for current service (spontaneous demand; Primary Care; emergency or hospital services; outpatient clinic; private services); delay to access the current service (up to 7 days; up to 30 days; up to 60 days; > 60 days); time attending the current service (up to 1 year; up to 3 years; up to 5 years; > 5 years); previous mental health care in Primary Care (did not receive; received); Community Health Agents (CHA) visits (does not receive; does receive); and social support perception (weak; regular; strong).

### Community Health Agents

Community Health Agents are professionals from the multidisciplinary team of Primary Care services, whose role is to develop actions to promote health and prevent diseases, focusing on educational activities in patients’ homes and community spaces. They are professionals with high school education, who receive training to work in a health territory that serves up to 750 people, and that, by definition, they also reside. In this way, the CHA are understood as the professionals who promote the integration of the Primary Care services and the Community.

### Statistical analysis

Statistical analyses were conducted using Stata 15 (Stata Corporation, College Station, Texas USA). In addition to their occurrence in the overall study population, the prevalence of both outcomes was calculated with an estimated 95% confidence interval for each of the covariates.

A weighting calibration procedure was used to reduce sample bias in relation to sex and age of the studied population. Sample weights were based on results obtained by a previous survey carried out between May and July 2019, as documented in service records.

Associations of timely care in Specialized Mental Health Services, and maintenance of a link with Primary Care with study covariates were tested using unadjusted and adjusted Poisson regression models with robust variance estimators. The adjusted analysis was carried out by the selection of confounders using forward stepwise selection among the study covariates. The selection criterion for inclusion was a p-value ≤ 0.20 [14].

Regarding the outcome ‘timely care in Specialized Mental Health Services’ the covariates selected as potential confounders were sex, schooling, per capita income, paid work status, diagnosis, identification of the need for mental health care, delay until first appointment, and first appointment service. Covariates were adjusted among themselves, and with each of the other covariates.

Regarding the outcome ‘maintenance of a link with Primary Care’, potential confounders were sex, ethnicity, paid work status, diagnosis, source of referral to the current service, previous mental health care in Primary Care, CHA visits, and social support. Similarly, they were adjusted among themselves, and with each of the other covariates.

### Ethical procedures

The study was approved by an accredited Ethics Committee, following the Brazilian regulatory standards and guidelines for research involving human beings (CNS Resolution 466/2012). It was similarly in accordance with the provisions of the Declaration of Helsinki.

## Results

### Characterization of participants

A total of 341 users of Specialized Mental Health Services answered the questionnaire of our study, of which 33.7% (n = 115) were from PCC-II, 34.0% (n =116) were from PCC-PSM, and 32.3% were from the Outpatient Clinic. The characterization of participants according to study variables is shown in Table 1. Variables with missing data were per capita income, delay until the first appointment, and delay to access the current service. Missing observations numbered 18, 19, and 11, respectively.

**Table 1:** Characterization of participants included in the study (n = 341)

	n	Prevalence (95% CI)
Sex		
Female	165	48.4 (43.1–53.7)
Male	176	51.6 (46.3–56.9)
Ethnicity		
White	190	55.7 (50.4–60.9)
Mixed race	119	34.9 (30.0–40.1)
Black	32	9.4 (6.7–12.9)
Age		
18–30	44	12.9 (9.5–16.9)
31–45	113	33.1 (28.3–38.3)
46–60	124	36.4 (31.4–41.6)
61+	60	17.6 (13.9–21.9)
Schooling		
0 to 4 years	65	19.1 (15.2–23.6)
5 to 8 years	144	42.2 (37.1–47.5)
9 + years	132	38.7 (33.7–44.0)
Paid work status		
Unemployed	264	77.4 (73.6–82.3)
Employed	77	22.6 (18.5–27.3)
Per capita income^a^		
< 0.5 minimum wage	80	24.8 (20.4–29.7)
0.5 to 1 minimum wage	155	48.0 (42.6–53.4)
> 1 minimum wage	88	27.2 (22.7–32.3)
Diagnosis		
Affective and neurotic disorders	122	35.8 (30.9–41.0)
Psychosis	74	21.7 (17.6–26.4)
Psychoactive substance misuse	83	24.3 (20.1–29.2)
As yet undiagnosed	62	18.2 (14.4–22.6)
Identification of the need for mental health care		
In a crises situation	127	37.2 (32.3–42.5)
Self diagnosis followed by spontaneous demand	95	27.9 (23.4–32.8)
Indicated by family and friends	74	21.7 (17.6–26.4)
In an appointment for general complaints	45	13.2 (10.0–17.2)
Delay until first appointment^b^		
Up to 7 days	116	36.0 (31.0–41.4)
Up to 30 days	75	23.3 (19.0–28.2)
Up to 90 days	52	16.1 (12.5–20.6)
Up to 1 year	37	11.5 (8.4–14.4)
>1 year	42	13.1 (10.0–17.2)
First appointment service		
Primary Care	45	13.2 (10.0–17.2)
Emergency or Hospital services	45	13.2 (10.0–17.2)
Outpatient Clinic	99	29.0 (24.5–34.1)
Psychosocial Care Centers	78	22.9 (18.7–27.6)
Private Services	74	21.7 (18.7–27.6)
Referral source for the current service		
Spontaneous demand	116	34.0 (29.2–39.2)
Primary Care	75	22.0 (17.9–26.7)
Emergency or Hospital services	38	11.1 (8.2–14.9)
Outpatient Clinic	77	22.6 (18.5–27.3)
Private Services	35	10.3 (7.5–13.9)
Delay to access the current service^c^		
Up to 7 days	161	48.8 (43.4–54.2)
Up to 30 days	99	30.0 (25.3–35.1)
Up to 60 days	37	11.2 (8.2–15.1)
> 60 days	33	10.0 (7.2–13.7)
Time attending the current service		
Up to 1 year	119	34.9 (30.0–40.1)
Up to 3 years	60	17.6 (13.9–22.0)
Up to 5 years	45	13.2 (10.0–17.2)
> 5 years	117	34.3 (29.5–39.5)
Previous mental health care in Primary Care		
Did not receive	212	62.2 (56.9–67.1)
Received	129	37.8 (32.8–43.1)
CHA visits		
Does not receive	206	60.4 (55.1–65.5)
Does receive	135	39.6 (34.5–44.9)
Social support perception		
Weak	59	17.3 (13.7–21.7)
Regular	137	40.2 (35.1–45.5)
Strong	145	42.5 (37.4–47.8)

^a^ n= 323

^b^ n= 322

^c^ n= 330

### Timely care in Specialized Mental Health Services

Information related to this outcome was obtained for 330 (96.8%) participants. The prevalence of timely care in Specialized Mental Health Services (i.e. the user having been attended within 15 days of referral) was 60.0% (95% CI 54–65). The prevalence of this outcome with respect to each of the variables included in the study, as well as unadjusted and adjusted relative risk values, are shown in Table 2.

**Table 2:** Unadjusted and adjusted^a^ associations between studied variables and timely access to care in Specialized Mental Health Services are provided with the use of Poisson regression models

	n	Prevalence (95% CI)	Unadjusted RR (95% CI)	Adjusted^a^ RR (95% CI)
Sex				
Female	160	47.5 (39.9–55.2)	1	1
Male	170	71.8 (64.6–78.0)	1.49 (1.21–1.83)***	1.13 (0.91–1.39)
Ethnicity				
White	184	54.9 (47.7–61.9)	1	1
Mixed race	115	67.8 (58.8–75.7)	1.31 (1.07–1.59)*	1.13 (0.95–1.36)
Black	31	61.3 (43.8–76.3)	1.08 (0.75–1.55)	0.82 (0.63–1.08)
Age				
18–30	43	69.8 (54.9–81.4)	1	1
31–45	110	65.4 (56.2–73.7)	0.91 (0.71–1.16)	0.98 (0.78–1.23)
46–60	119	59.7 (50.7–68.0)	0.84 (0.66–1.08)	0.95 (0.73–1.23)
61+	58	43.1 (31.2–55.9)	0.61 (0.43–0.88)*	0.87 (0.60–1.26)
Schooling				
0 to 4 years	64	78.1 (66.6–86.5)	1	1
5 to 8 years	138	60.9 (52.5–68.6)	0.86 (0.69–1.07)	0.89 (0.70–1.13)
9+ years	128	50.0 (41.5–58.5)	0.66 (0.51–0.86)*	0.79 (0.60–1.03)
Paid work status				
Unemployed	253	60.5 (54.3–66.3)	1	1
Employed	77	58.4 (47.3–68.8)	1.05 (0.84–1.31)	1.15 (0.93–1.41)
Per capita income^b^				
< 0.5 minimum wage	78	66.7 (55.6–76.1)	1	1
0.5 to 1 minimum wage	151	61.6 (53.6–68.9)	0.96 (0.77–1.19)	0.82 (0.66–1.03)
> 1 minimum wage	86	51.2 (40.8–61.4)	0.72 (0.54–0.98)*	0.74 (0.55–0.99)*
Diagnosis				
Affective and neurotic disorders	119	37.8 (29.6–46.8)	1	1
Psychosis	69	68.1 (56.4–77.9)	1.77 (1.30–2.40)***	1.47 (1.06–2.04)*
Psychoactive substance misuse	80	82.5 (72.7–89.3)	2.18 (1.67–2.84)***	1.70 (1.24–2.32)**
As yet undiagnosed	40	64.5 (52.1–75.3)	1.62 (1.17–2.24)*	1.31 (0.93–1.83)
Identification of the need for mental health care				
In a crises situation	124	66.1 (57.4–73.9)	1	1
Self diagnosis followed by spontaneous demand	93	55.9 (45.8–65.6)	0.77 (0.60–0.99)*	0.86 (0.68–1.08)
Indicated by family and friends	70	71.4 (59.9–80.7)	1.07 (0.87–1.32)	1.08 (0.85–1.36)
In an appointment for general complaints	43	32.6 (20.5–47.5)	0.44 (0.27–0.74)*	0.53 (0.32–0.87)*
Delay until first appointment^c^				
Up to 7 days	114	80.7 (72.5–86.9)	1	1
Up to 30 days	74	50.0 (38.9–61.1)	0.56 (0.42–0.74)***	0.70 (0.53–0.93)*
Up to 90 days	51	39.2 (27.0–52.9)	0.46 90.31–0.68)***	0.46 (0.31–0.68)***
Up to 1 year	34	29.4 (16.8–46.2)	0.42 (0.24–0.73)*	0.43 (0.25–0.74)*
> 1 year	40	75.0 (59.8–85.8)	0.93 (0.75–1.15)	0.79 (0.61–1.01)
First appointment service				
Primary Care	45	40.0 (27.0–54.5)	1	1
Emergency or Hospital services	44	59.1 (44.4–72.3)	1.34 (0.83–2.16)	1.00 (0.62–1.59)
Outpatient Clinic	94	50.0 (40.1–59.9)	1.16 (0.75–1.80)	1.42 (0.94–2.13)
Psychosocial Care Centers	76	84.2 (74.4–90.7)	2.13 (1.45–3.13)***	1.33 (0.89–1.97)
Private Services	71	60.6 (48.9–71.1)	1.39 (0.90–2.15)	1.13 (0.73–1.76)
Referral source for the current service				
Spontaneous demand	110	72.7 (63.7–80.2)	1	1
Primary Care	74	35.1 (25.2–46.5)	0.51 (0.36–0.72)***	0.61 (0.40–0.93)*
Emergency or Hospital services	37	48.6 (33.4–64.1)	0.52 (0.41–0.94)*	0.86 (0.57–1.29)
Outpatient Clinic	74	68.9 (57.7–78.3)	0.99 (0.82–1.21)	1.01 (0.79–1.28)
Private Services	35	65.7 (49.1–79.1)	0.85 (0.63–1.17)	0.84 (0.63–1.13)
Time attending the current service				
Up to 1 year	114	69.3 (60.3–77.0)	1	1
Up to 3 years	59	50.8 (38.4–63.2)	0.71 (0.52–0.98)*	0.75 (0.58–0.98)*
Up to 5 years	43	48.8 (34.6–63.2)	0.66 (0.45–0.97)*	0.80 (0.54–1.19)
>5 years	114	59.6 (50.5–68.2)	0.87 (0.71–1.08)	1.06 (0.82–1.38)

Relative risks (RR) with 95% corresponding confidence intervals (CIs) are presented (n = 330)

^*^P ≤ 0.05, ^**^P ≤ 0.01, ^***^P ≤ 0.001

^a^ Adjusted by sex; schooling; per capita income; diagnosis; identification of the need for mental health care; delay until first appointment; first appointment service

^b^ n = 323

^c^ n = 322

There was evidence of an association of timely care in Specialized Mental Health Services and the diagnosis of psychosis (RR: 1.47; 95% CI 1.06, 2.04) and psychoactive substance misuse (RR: 1.70; 95% CI 1.24, 2.32).

At the same time, we identified an inverse association of timely care in Specialized Mental Health Services with the following outcomes: higher per capita income (RR: 0.74; 95% CI 0.55, 0.99); having their need for mental health care identified in an appointment for general complaints (RR: 0.53; 95% CI 0.32, 0.87); having been referred to the current service by Primary Care (RR: 0.61; 95% CI 0.40, 0.93); and having attended the current service for up to 3 years.

Delay until the first appointment was also inversely associated with timely care in Specialized Mental Health Services. Except for those who took more than one year to attend their first appointment, patients who waited more than 7 days to be attended demonstrated decreased risk of the outcome in comparison to those waiting up to 30 days (RR: 0.70; 95% CI 0.53, 0.93), up to 90 days (RR: 0.46; 95% CI 0.31, 0.68), or up to one year (RR: 0.43; 95% CI 0.25, 0.74).

### Maintenance of a link with Primary Care

Information related to this outcome was obtained for all participants. The prevalence of maintaining a link with Primary Care (i.e. having attended Primary Care services at least once in the last 6 months) was 56.9% (95% CI 51–62) for the whole sample. The prevalence of this outcome by study covariate, as well as unadjusted and adjusted relative risks, are given in Table 3.

**Table 3:** Unadjusted and adjusted^a^ associations between studied variables and maintaining the link with Primary Care are provided with the use of Poisson regression models. Relative risks (RR) with 95% corresponding confidence intervals (CIs) are presented (n=330)

	n	Prevalence (95%CI)	Unadjusted RR (95%CI)	Adjusted^a^ RR (95%CI)
Sex				
Female	165	69.1 (61,8–75,6)	1	1
Male	176	45.4 (38.3–52.8)	0.66 (0.53–0.82)***	0.82 (0.67–0.99)*
Ethnicity				
White	190	59.5 (52.4–66.2)	1	1
Mixed race	119	58.8 (49.8–67.3)	0.96 (0.77–1.19)	0.95 (0.78–1.16)
Black	32	34.4 (20.4–51.6)	0.69 (0.43–1.11)	0.80 (0.50–1.29)
Age				
18–30	44	50.0 (34.8–64.2)	1	1
31–45	113	53.1 (0.43–0.62)	1.10 (0.78–1.55)	1.00 (0.75–1.34)
46–60	124	60.5 (51.7–68.6)	1.22 (0.87–1.69)	1.09 (0.83–1.44)
61+	60	61.7 (49.0–72.9)	1.23 (0.86–1.77)	0.97 (0.71–1.33)
Schooling				
0 to 4 years	65	60.0 (47.9–71.0)	1	1
5 to 8 years	144	58.3 (50.2–66.1)	0.95 (0.72–1.24)	1.05 (0.80–1.37)
9+ years	132	53.8 (45.3–62.1)	0.94 (0.71–1.23)	0.99 (0.76–1.30)
Paid work status				
Unemployed	264	60.2 (54.2–65.9)	1	1
Employed	77	45.4 (34.8–56.5)	0.74 (0.55–0.99)*	0.78 (0.60–1.00)*
Per capita income^b^				
<0.5 minimum wage	80	61.2 (50.3–71.2)	1	1
0.5 to 1 minimum wage	155	54.8 (47.0–62.5)	0.89 (0.69–1.15)	0.91 (0.73–1.14)
>1 minimum wage	88	61.4 (50.9–70.9)	1.05 (0.80–1.37)	1.09 (0.85–1.40)
Diagnosis				
Affective and neurotic disorders	122	72.1 (63.6–79.3)	1	1
Psychosis	74	55.4 (44.1–66.2)	0.66 (0.50–0.87)*	0.69 (0.52–0.91)*
Psychoactive substance misuse	83	36.1 (26.6–46.9)	0.47 (0.33–0.66)***	0.57 (0.41–0.80)**
As yet undiagnosed	62	56.4 (44.1–68.1)	0.76 (0.58–0.98)*	0.84 (0.66–1.07)
Identification of the need for mental health care				
In a crises situation	127	59.1 (50.4–67.2)	1	1
Self diagnosis followed by spontaneous demand	95	55.8 (45.8–65.4)	1.01 (0.78–1.30)	0.92 (0.72–1.17)
Indicated by family and friends	74	45.9 (35.1–57.2)	0.84 (0.61–1.15)	0.86–0.65–1.15)
In an appointment for general complaints	45	71.1 (56.6–82.3)	1.26 (0.97–1.65)	1.09 (0.83–1.44)
Delay until first appointment^c^				
Up to 7 days	116	54.3 (45.2–63.1)	1	1
Up to 30 days	75	65.3 (54.0–75.1)	1.19 (0.92–1.55)	1.00 (0.78–1.27)
Up to 90 days	52	53.8 (40.5–66.7)	1.02 (0.74–1.41)	0.93 (0.69–1.25)
Up to 1 year	37	54.0 (38.4–69.0)	1.04 (0.72–1.50)	0.83 (0.59–1.17)
>1 year	42	54.8 (39.9–68.8)	1.00 (0.70–1.41)	1.07 (0.77–1.47)
First appointment service				
Primary Care	45	71.1 (56.6–82.3)	1	1
Emergency or Hospital services	45	48.9 (35.0–63.0)	0.62 (0.42–0.92)*	1.09 (0.72–1.66)
Outpatient Clinic	99	66.7 (56.9–75.2)	0.86 (0.67–1.09)	1.19 (0.90–1.57)
Psychosocial Care Centers	78	43.6 (33.1–54.6)	0.55 (0.39–0.78)**	1.10 (0.75–1.61)
Private Services	74	54.0 (42.8–64.9)	0.76 (0.57–1.01)	1.19 (0.88–1.61)
Referral source for the current service				
Spontaneous demand	116	43.1 (34.4–52.2)	1	1
Primary Care	75	73.3 (62.4–82.0)	1.75 (1.32–2.30)***	1.38 (1.06–1.79)*
Emergency or Hospital services	38	55.3 (19.7–69.8)	1.38 (0.95–2.02)	1.17 (0.81–1.68)
Outpatient Clinic	77	59.7 (48.6–70.0)	1.39 (1.01–1.90)*	1.31 (0.98–1.75)
Private Services	35	62.9 (46.3–76.8)	1.57 (1.11–2.23)*	1.66 (1.19–2.32)*
Delay to access the current service^d^				
Up to 7 days	161	47.2 (39.6–54.9)	1	1
Up to 30 days	99	66.7 (56.9–75.2)	1.50 (1.18–1.90)**	1.04 (0.81–1.34)
Up to 60 days	37	64.9 (48.8–78.2)	1.39 (1.00–1.93)*	1.07 (0.79–1.45)
>60 days	33	69.7 (52.6–82.6)	1.55 (1.14–2.11)*	0.93 (0.67–1.29)
Time attending the current service				
Up to 1 year	119	50.4 (41.6–59.2)	1	1
Up to 3 years	60	45.0 (33.1–57.5)	0.84 (0.59–1.21)	0.84 (0.62–1.15)
Up to 5 years	45	57.8 (43.3–71.0)	1.13 (0.82–1.56)	1.00 (0.72–1.38)
>5 years	117	69.2 (60.4–76.9)	1.22 (0.96–1.55)	1.11 (0.89–1.39)
Previous mental health care in Primary Care				
Did not receive	212	48.6 (41.9–55.3)	1	1
Received	129	70.5 (62.2–77.7)	1.47 (1.21–1.79)***	1.12 (0.92–1.38)
CHA visits				
Does not receive	206	49.0 (42.3–55.8)	1	1
Does receive	135	68.9 (60.6–76.1)	1.37 (1.12–1.67)*	1.26 (1.04–1.53)*
Social support perception				
Weak	59	76.3 (64.0–85.3)	1	1
Regular	137	56.9 (48.6–64.9)	0.73 (0.58–0.91)*	0.80 (0.65–0.99)*
Strong	145	49.0 (41.0–57.0)	0.62 (0.49–0.89)***	0.73 (0.58–0.91)*

^*^P ≤ 0.05, ^**^P ≤ 0.01, ^***^P ≤ 0.001

^a^ Adjusted by sex; ethnicity; paid work status; diagnosis; referral source to the current service; previous mental health care in Primary Care; CHA visits; social support

^b^ n = 323

^c^ n = 322

^d^ n = 330

We identified evidence of an association between the outcome ‘maintenance of a link with Primary Care’ and the referral for current service by Primary Care (RR: 1.38; 95% CI 1.06, 1.79) and Private Services (RR: 1.66; 95% CI 1.19, 2.32). There was also an association between the outcome and receiving visits from Community Health Agents (CHA) (RR: 1.26; 95% CI 1.04, 1.53).

In contrast, an inverse association was found between the outcome and male sex (RR: 0.82; 95% CI 0.67, 0.99), being employed (RR: 0.78; 95% CI 0.60, 1.00), having a diagnosis of psychosis (RR: 0.69; 95% CI 0.52, 0.91) or psychoactive substance misuse (RR: 0.57; 95% CI 0.41, 0.80), and a greater perception of social support: regular (RR: 0.80; 95% CI 0.65, 0.99), or strong (RR: 0.73; 95% CI 0.58, 0.91).

## Discussion

This is the first study to investigate the determinants of timely access to Specialized Mental Health Services and maintenance of a link with Primary Care. Such work augments the current evidence–base and provides further evidence for understanding the factors that affect patient’s experiences in a complex mental health network with different forms of access and the aspects that foster continuity of care.

While being referred by Primary Care to the specialized service was negatively associated with timely access, this was positively associated with maintaining the link with Primary Care. Also, in relation to the diagnosis, it can be observed that patients diagnosed with psychosis and psychoactive substance misuse were more likely to access specialized services in a timely manner, however, they were less likely to maintain the link with Primary Care. These findings contribute to the global discussion about two important challenges for better mental health care, the low integration between Primary Care services and specialized services [15] and the barriers of access that individuals with mental health and/or substance use issues face in Primary Care [16].

Previous studies have also indicated that Primary Care contact was associated with longer delays accessing specialized mental health care [1, 17]. However, at the same time, this type of contact was associated with better pathways to care, fewer contacts with emergency services, and greater adherence to specialized services [17]. It appears, therefore, that the need for training Primary Care workers in the detection and management of mental health cases should be reinforced, as should be efforts to integrate the mental health care network.

Globally, several efforts have been made to establish greater integration between specialized services and Primary Care. In countries such as the United Kingdom, Spain and Canada, initiatives such as shared or collaborative care aims to link professionals and to develop strategies to collect and share information on the progress of patients [10].

It should be considered that in addition to enhancing the identification and management of mental health cases, the strengthening of this strategies could help in solving another major problem of the mental health care networks; namely, the low return of users to Primary Care levels. Previous studies have suggested that continuity of care is a critical issue when referring patients from specialized care back to Primary Care, as few people appear to reach Primary Care centers after referral [4, 19].

In a study conducted in the United States with patients of a community mental health center [18], similar to our results, 41% of patients did not attend Primary Care in the six months prior to the survey. In the same study, 63% of patients were unable to identify a Primary Care provider by name and 14% reported using the emergency department for routine care.

Besides problems related to the continuity of mental health treatment, low contact with Primary Care appears to be a main contributor to the mortality gap experienced by people living with mental disorders worldwide [19]. It is estimated that this population experiences mortality rates two to three times higher than the general population, with life expectancy reduced by 10–30 years [20]. Among the factors contributing to these outcomes, there is a high prevalence in the population of hypertension, diabetes, heart disease and other conditions that could be treated by Primary Care, if identified in a timely manner [21, 22].

Our results still raise an important discussion about the stigma related to psychosis and substance use. Both the greater absorption of these patients by specialized services and the lower likelihood that they will access Primary Care after being admitted to specialized services may be related to stigma. A study conducted in the United States [23] comparing professionals at both Primary Care and secondary healthcare centres found that physicians and nurses at Primary Care had more negative attitudes toward people with psychosis than their colleagues at secondary healthcare centres.

In relation to substance use, in addition to stigma, other social issues must be observed. Studies in Latin America [24, 25] have shown that although some patients occasionally access the Primary Care to obtain clinical health care, the substance use is not brought up. The professionals in turn avoid talking about the subject because they do not wish to be mistaken as informants for the police or drug dealers [24].

In relation to the action of Primary Care, our results emphasize the importance of non-medical professionals in promoting the continuity of care. Among these professionals are the CHA, whose visits to users were associated with maintaining a link with Primary Care. Through home visits, these professionals are responsible for collecting information regarding the population’s health needs, identifying users with health problems, and referring them to the health unit [26]. Despite the importance of their role, however, they are often overlooked in the discussion of mental health cases management. This lack of recognition relative to other professions may stem from educational bias, as many CHA are individuals with a high school education [10].

Finally, we highlight that our results suggest that patients’ first steps in seeking help may influence other aspects of their trajectory within the health system. With the exception of those who attended their first mental health consultation after waiting more than a year, patients who waited more than 7 days to be treated were less likely to access specialized services in a timely manner (as compared to those who waited up to 7 days for treatment). These results are consistent with a review regarding the pathways to mental health care among young people [27], where the service responses to help-seeking were important determinants of patients’ pathways.

Some limitations should be considered in the interpretation of the present results. This is a cross-sectional study; therefore, reverse causality cannot be ruled out. Also, many variables were measured retrospectively, and thus are subject to recall error and bias. Additionally, it should be highlighted that this study recruited users who had access to and remained linked to specialized outpatient mental health services. Those who had previously discontinued care or did not have access to these services were therefore not included in our sample. Thus, in the city from which we drew our sample, the present results may not be representative of the full population with mental health disorders and their experiences in the mental health network.

## Conclusions

Our study is an essential step towards formulating policies that ensure easier, timelier access to care and, thereby, shaping mental health patients’ outcomes. In addition to punctuating the determinants for timely access, our study also punctuates the determinants of maintaining a link with Primary Care, which is essential to bridge the mortality gap and health disparities experienced by this population worldwide.

We highlight the need to strengthen communication between Primary Care and specialized services as a measure both to qualify access in a timely manner and to promote continuity of care. In this context, the role of non-medical professionals should be highlighted, since the action of Community Health Agents has proved to be effective in fostering the link with Primary Care.

Another relevant finding is the disparity faced by patients diagnosed with psychosis and substance use issues, which may be related to the stigma attached to these diagnoses. In this sense, we reinforce the need to establish anti-stigma policies and programs, especially in Primary Care, which must be able to receive this population as a gateway to the health system.

## Data Availability

The datasets used and/or analysed during the current study are available from the corresponding author on reasonable request.

## Abbreviations

CHA: Community Health Agents
PCC-II: Psychosocial Care Center II
PCC-PSM: Psychosocial Care Center for Psychoactive Substance Misuse
SMHS: Specialized Mental Health Services

## References

[1] Volpe U, Mihai A, Jordanova V, Sartorius N (2015). The pathways to mental healthcare worldwide. Curr Opin Psychiatry.

[2] Evans-Lacko S, Jarrett M, McCrone P, Thornicroft G (2008). Clinical pathways in psychiatry. Br J Psychiatry.

[3] Gater R, Almeida E, Sousa D, Barrientos G, Caraveo J, Chandrashekar C, Dhadphale M (1991). The pathways to psychiatric care: a cross-cultural study. Psychol Med.

[4] Amaral C, Onocko-Campos R, de Oliveira P, Pereira M, Ricci E, Pequeno M (2018). Systematic review of pathways to mental health care in Brazil: narrative synthesis of quantitative and qualitative studies. Int J Ment Health Syst.

[5] Global Burden of Disease Collaborative Network. Global Burden of Disease Study 2016 (G2016) Results. http://ghdx.healthdata.org/gbd-results-tool. 2016. Accessed 17 Feb 2020

[6] World Health Organization (2017). Depression and Other Common Mental Disorders: Global Health Estimates.

[7] Onocko-Campos R, Amaral C, Saraceno B, de Oliveira B, Treichel C, Delgado P. Atuação dos Centros de Atenção Psicossocial em quatro centros urbanos no Brasil. Revista Panamericana De Salud Pública. 2018. 10.26633/rpsp.2018.113 10.26633/RPSP.2018.113 PMC6386118 31093141

[8] Wang Y, Chiavegatto-Filho A, Campanha A, Malik A, Mogadouro M, Cambraia M (2016). Patterns and predictors of health service use among people with mental disorders in São Paulo metropolitan area, Brazil. Epidemiology and Psychiatric Sciences.

[9] Vannucchi A, Carneiro Junior N (2012). Modelos tecnoassistenciais e atuação do psiquiatra no campo da atenção primária à saúde no contexto atual do Sistema Único de Saúde. Brasil. Physis: Revista De Saúde Coletiva.

[10] Treichel C, Campos R, Campos G (2019). Impasses e desafios para consolidação e efetividade do apoio matricial em saúde mental no Brasil. Interface - Comunicação, Saúde, Educação,.

[11] De Hert M, Correll C, Bobes J, Cetkovich-Bakmas M, Cohen D, Asai I (2011). Physical illness in patients with severe mental disorders. I. Prevalence, impact of medications and disparities in health care. World Psychiatry.

[12] Ferrari M, Flora N, Anderson K, Haughton A, Tuck A, Archie S (2016). Gender differences in pathways to care for early psychosis. Early Intervention in Psychiatry.

[13] IBGE – Instituto Brasileiro de Geografia e Estatística. Estimativas de População. https://www.ibge.gov.br/estatisticas/sociais/populacao/. 2019. Accessed 17 Feb 2020.

[14] Maldonado G, Greenland S (1993). Simulation Study of Confounder-Selection Strategies. American Journal of Epidemiology.

[15] Petersen I, van Rensburg A, Kigozi F, Semrau M, Hanlon C, Abdulmalik J (2019). Scaling up integrated primary mental health in six low- and middle-income countries: obstacles, synergies and implications for systems reform. Bjpsych Open.

[16] Ross L, Vigod S, Wishart J, Waese M, Spence J, Oliver J et al. Barriers and facilitators to primary care for people with mental health and/or substance use issues: a qualitative study. BMC Fam Practice. 2015. 10.1186/s12875-015-0353-3 10.1186/s12875-015-0353-3 PMC4604001 26463083

[17] Anderson K, Fuhrer R, Schmitz N, Malla A (2012). Determinants of negative pathways to care and their impact on service disengagement in first-episode psychosis. Soc Psychiatry Psychiatr Epidemiol.

[18] Miller CL, Druss B, Dombrowski E, Rosenheck R (2003). Barriers to Primary Medical Care Among Patients at a Community Mental Health Center. Psychiatric Services.

[19] World Health Organization. MhGAP intervention guide for mental, neurological and substance use disorders in non-specialized health settings: Mental Health Gap Action Programme (mhGAP). WHO: Geneva; 2010. 23741783

[20] Cook J, Razzano L, Swarbrick M, Jonikas J, Yost C, Burke L (2015). Health Risks and Changes in Self-Efficacy Following Community Health Screening of Adults with Serious Mental Illnesses. PLOS ONE.

[21] Woodhead C, Ashworth M, Schofield P, Henderson M (2014). Patterns of physical co-/multi-morbidity among patients with serious mental illness: a London borough-based cross-sectional study. BMC Family Practice.

[22] Baller J, McGinty E, Azrin S, Juliano-Bult D, Daumit G (2015). Screening for cardiovascular risk factors in adults with serious mental illness: a review of the evidence. BMC Psychiatry.

[23] Mittal D, Corrigan P, Sherman M, Chekuri L, Han X, Reaves C (2014). Healthcare providers’ attitudes toward persons with schizophrenia. Psychiatr Rehab J.

[24] Paula ML, Jorge MSB, Vasconcelos MGF, Albuquerque RA (2014). Assistance to the drug user in the primary health care. Psicologia em Estudo.

[25] Filho AJT (2016). Approach to drug use in Primary Care – an awareness. Revista Médica de Minas Gerais.

[26] Waidman MAP, Costa B, Paiano M (2012). Percepções e atuação do Agente Comunitário de Saúde em saúde mental. Revista da Escola de Enfermagem da USP.

[27] MacDonald K, Fainman-Adelman N, Anderso K, Iyer S (2018). Pathways to mental health services for young people: a systematic review. Soc Psychiatry Psychiatric Epidemiol.

